# Adherence to the Mediterranean Diet in Spanish University Students: Association with Lifestyle Habits, Mental and Emotional Well-Being

**DOI:** 10.3390/nu17040698

**Published:** 2025-02-15

**Authors:** Gloria Tomás-Gallego, Josep María Dalmau-Torres, Raúl Jiménez-Boraita, Javier Ortuño-Sierra, Esther Gargallo-Ibort

**Affiliations:** 1 Department of Didactics of Body Expression, University of La Rioja, 26006 Logroño, Spain; jose-maria.dalmau@unirioja.es (J.M.D.-T.); esther.gargallo@unirioja.es (E.G.-I.); 2 Department of Didactic of Physical Education and Health, Universidad Internacional de La Rioja, 26006 Logroño, Spain; raul.jimenez@unir.net; 3 Department of Educational Sciences, University of La Rioja, 26006 Logroño, Spain; javier.ortuno@unirioja.es

**Keywords:** Mediterranean diet, health, university students, lifestyle, physical activity, emotional intelligence, healthy habits, mental health

## Abstract

**Background**: The Mediterranean Diet is recognized as one of the healthiest dietary patterns; however, in recent years, a decline in adherence has been observed in Mediterranean countries. University students represent a particularly vulnerable population, as starting university introduces new influences and responsibilities that directly impact their lifestyle and health. **Objective**: Analyze adherence to the Mediterranean Diet among university students and its association with other lifestyle habits and mental and physical health indicators. **Methods**: A cross-sectional study was conducted with a sample of 1268 students (23.65 ± 7.84 years) from a university in northern Spain between November 2020 and March 2021. An online questionnaire was administered to assess Mediterranean Diet adherence along with variables such as perceived stress, self-esteem, life satisfaction, suicidal behavior, emotional and behavioral problems, emotional intelligence, physical activity, sedentary behavior, alcohol consumption, and compulsive internet use. **Results**: 29.26% of students had high adherence to the Mediterranean Diet. Regression analysis indicated that high adherence was associated with higher levels of emotional intelligence, as well as lower levels of suicidal ideation, emotional problems, and compulsive internet use. **Conclusions**: The associations found between Mediterranean Diet and other indicators and lifestyle habits highlight the need for interdisciplinary promotion strategies within the university ecosystem.

## 1. Introduction

A person’s diet is influenced by socio-economic and personal factors that interact and shape individual dietary habits, which vary over time. These factors range from economic constraints, such as income and food prices, personal preferences and beliefs, cultural traditions, as well as political, geographic and environmental influences. Promoting a healthy nutritional environment, therefore, requires the public and private sectors to work together [1]. As an ecosystem in its own right, universities play a fundamental role in this context, not only through intervention, education and research within their community, but also by impacting society as a whole. As such, nutrition is one of the key pillars of health promotion programmes on university campuses, which is tackled from a holistic developmental perspective [2,3].

The Mediterranean Diet (MD) is regarded as one of the healthiest dietary patterns since it was first identified by Keys et al. [4] and up to the present day [5]. Mediterranean diets are typically rich in fruit and vegetables and imply a responsible consumption of meat, fish, olive oil, dairy products and seeds, along with other types of food. This is the traditional Mediterranean diet [6,7]. Research has focused on adherence to the MD, given its role as a protective factor for physical, physiological, mental, and socio-emotional health [5,8]. Literature suggests that the higher the adherence to the MD, the better the overall health and vitality, and it is considered both a pre-emptive and a treatment medical recommendation [9]. Furthermore, increased adherence to the MD reduces cardiovascular disease, prevents several types of cancer and improves emotional and cognitive functioning, among other benefits [10,11]. Recent studies on cognitive decline, dementia and Alzheimer’s disease highlight that observing a Mediterranean dietary pattern throughout life contributes to preventing and delaying its onset [12,13]. Moreover, data presented by Antonopoulou’s research team [14] suggest that maintaining good eating habits improves academic performance.

Research has shown that individuals who adhere to the MD more closely are more physically fit, both in terms of muscle and cardiorespiratory fitness [15]. Accordingly, the sedentary lifestyle shows an inverse relationship, whereby those with high adherence to the MD have lower rates of sedentary behaviour [16]. In addition, unhealthy consumption is also inversely related to adherence to the MD. As a result, the greater adherence to the MD, the less compulsive internet use [17] and alcohol abuse [18], the latter being closely linked to eating disorders and the use of food as an aggravating factor for the effects of alcohol, phenomenon known as “drunkorexia” or “food and alcohol disturbance” (FAD) [19].

Nutritional habits are among the first aspects to change throughout life stages or in response to personal experiences and circumstances, despite the demonstrated benefits of keeping healthy eating habits [20]. Diet is closely linked to a person’s psychological and emotional state, making it a key factor in mental health [21]. The transition to adulthood seems to represent one of the critical junctures when it comes to establishing healthy nutritional habits, as it is at this time that people begin to make their own decisions [22]. University students are at this vital moment emerging adults who become part of a vulnerable population when it comes to adopting healthy habits. This vulnerability is caused by the numerous changes they must face in a short period of time, such as economic and housing independence by living outside the family unit [23]. They often move from parental or guardian-managed food organisation to their self-management and decision-making. It appears that this is not only the case for students who leave home and become independent but also for others, as they have to adjust to class schedules, which often require them to eat on the university campus [24]. As a result, low adherence to the MD is observed in the first year of university, as shown in the studies by Cobo-Cuenca [15] and Castro-Cuesta [25], in which 65% and 55% of Spanish students, respectively, show poor adherence to the MD. The new eating patterns chosen by young people are driven by convenience and time constraints, together with a lack of shopping experience, the wide range of ready meals on offer and economic concerns [15,26,27]. This adherence has even been shown to decrease, and nutritional habits worsen as university courses advance [28,29].

The situation of Spanish university students is similar to that discussed in the previous paragraphs. The recent research by Muñoz et al. [30] shows that Spanish university students are increasingly moving away from the MD. Indreica et al. reported higher MD scores for Spanish compared to Romanian university students [31]. However, only 20–30% of Spanish university students had good DM adherence [15,25,32].

Therefore, this study focused on analysing adherence to the MD among university students. The primary objective of the research was to assess adherence to the MD among students at a university in the north of Spain. The second objective was to analyse the association between students’ other lifestyle habits and their mental and emotional well-being. To do so, the following variables were measured: perceived stress, self-esteem, life satisfaction, suicidal behaviour, emotional and behavioural issues, emotional intelligence, physical activity, sedentary lifestyle, alcohol consumption, and compulsive internet use.

## 2. Materials and Methods

### 2.1. Study Design and Participants

During the 2020–2021 academic year, the University of La Rioja enrolled a total of 4408 students, which were distributed between the faculties and its two higher education centres. Convenience sampling was carried out in the different faculties and study years to gather the student sample. Students enrolled in e-learning and/or who did not understand Spanish (e.g., exchange students) were excluded. This resulted in a study population of 4259 students.

An initial sample of 2200 students, 52% of the population, was recruited for this study. Incomplete questionnaires and questionnaires containing random, pseudo-random or dishonest, according to the Oviedo Infrequency Scale (INF-OV) [33], answers were filtered out and eliminated, leaving a final sample of 1268 students (823 females and 445 males). The mean age was 23.65 years (SD = 7.84) ranging from 17 to 80 years. For this study, two groups were formed according to age, following Arnett’s social theory [23]: group 1, considered “emerging adults”, with an age range between 17 and 25 years old (80.5%); and group 2, considered “adults”, with n = 247 and an age range between 26 and 80 years old (19.5%).

### 2.2. Procedure

A pretesting phase was carried out with a pilot sample of 25 university students before data collection. These students were not part of the final study sample. The aim of this phase was to assess the clarity of the questions, the comprehension of the items, the time needed and the functionality of the online survey. Following the pre-test phase, minor adjustments were made to the wording of some items to improve comprehension, particularly in the sections concerning alcohol consumption and physical activity.

Participants were sent a link to the final survey tool via their university email address. This tool is an online survey carried out on the SurveyMonkey platform. The Vice-Rector’s Office for Students approved the distribution of the survey and provided the students’ email addresses. The survey was answered individually and from any device with an Internet connection (mobile phone, tablet or PC). Only one response per student was possible.

The University Ethics Committee approved the survey tool and the study. Before starting the survey and participating in the study, respondents were required to give their consent after reading all the relevant information. Confidentiality and anonymity of the participants’ data were strictly maintained throughout the research process. Responses were collected between November 2020 and March 2021.

### 2.3. Instruments

A research tool was created by compiling eleven validated tests and questionnaires together with socio-demographic questions. The tests and questionnaires used are specified below.

#### 2.3.1. Adherence to the MD

The KIDMED questionnaire was chosen to measure adherence to the MD, in its Spanish version validated in young people [34]. This has sixteen items to assess whether inherent patterns of MD adherence are being followed or not. Responses are dichotomous (yes or no) and are coded as one or minus one. All items are added together to obtain the final score, which can range between four and twelve points. Therefore, three cut-off points are produced, classifying results above eight points as “optimal Mediterranean diet or high adherence”, between four and seven points as “Mediterranean diet to be improved or medium adherence”, and finally, equal to or below three points, as “low adherence”. This questionnaire has a Kappa value reliability ranging from 0.504 to 0.849 [35].

#### 2.3.2. Physical Activity

The short Spanish version of the International Physical Activity Self-Report Questionnaire (IPAQ-SF) [36,37] measured physical activity and sedentary habits, which has been previously validated in Spanish university students [38]. This questionnaire examines the intensity and type of physical activity performed in the seven days prior to taking the questionnaire, including vigorous, moderate, and walking and sitting time. Frequency and duration are recorded for each type of activity. Reliability scores are above 0.65 on Spearman’s correlation coefficient [37]. Total scores are calculated by adding duration and frequency. The volume of physical activity is calculated in METs (metabolic equivalent). Moreover, this tool assesses sedentary behaviour through a single dichotomous item. This item considers respondents to have a sedentary lifestyle if they report more than six hours of sedentary activities per day.

#### 2.3.3. Alcohol Consumption

The Spanish validated version of AUDIT for university students [39] was used to identify alcohol consumption patterns. The World Health Organization (WHO) developed this test with ten questions on the amount, frequency and consequences of alcohol consumption. All item scores are added together so as to determine whether there is high-risk drinking or dependent drinking behaviour. The values range from zero to 40 points, and the higher the value, the greater the risk. As for the reliability of the Spanish version in university students, the Spearman correlation coefficient was 0.87.

#### 2.3.4. Compulsive Internet Use

The Spanish version of the Compulsive Internet Use Scale (CIUS) [40], was used to analyse problematic Internet use, validated by Ortuño et al. [41] in the Spanish population. The scale measures five dimensions: loss of control (items 1, 2, 5, and 9), worry (items 4, 6, and 7), withdrawal symptoms (item 14), coping or mood alteration (items 12 and 13), and interpersonal and intrapersonal conflict (items 3, 8, 10, and 11). The scale has 14 items that are scored on a five-point Likert scale and the total scores are obtained by adding them all together. This scale has a reliability level equal to 0.91 on the McDonald’s Omega [41].

#### 2.3.5. Emotional and Behavioural Problems

Goodman’s [42] Strengths and Difficulties Questionnaire (SDQ) was used to assess emotional and behavioural variables. The self-report version for adults (+18) validated in Spanish was used (https://www.sdqinfo.org/py/sdqinfo/b3.py?language=Spanish) (accessed on 20 January 2020). There are 25 questions grouped into five subscales: emotional distress, behavioural issues, peer conflict, hyperactivity and prosocial behaviour. Each subscale consists of five items that are answered on a three-point Likert scale. The sum of the individual subscale scores, with the exception of the prosocial subscale, produces the total difficulties score. This questionnaire showed acceptable levels of reliability, α = 0.84 for the Total Difficulties score, and ranged between 0.71 and 0.75 for the subscales [43].

#### 2.3.6. Emotional Intelligence

The Trait Meta-Mood Scale (TMMS) was used to measure emotional intelligence, in its abbreviated Spanish version [44] with 24 items. It is a self-report tool originally designed by Salovey’s team [45], with a reliability above 0.85 [46], which assesses three cognitive construct components of emotional intelligence: attention to feelings, emotional clarity, and emotion repair. Possible responses are given on a five-point Likert scale, with one being “strongly disagree” to five being “strongly agree”. Scores are calculated for each component individually and range from eight to 40 points. The results are classified according to whether the respondent is male or female.

#### 2.3.7. Self-Esteem

The Rosenberg Self-Esteem Scale (RSES) [47] assessed students’ self-esteem. The validated Spanish translation [48] was used. It measured respondents’ general perceptions of their own self-esteem and self-worth. It is unidimensional and consists of 10 items, five positive and five negative, which are scored using a four-point Likert-type scale, from 1 = strongly disagree to 4 = strongly agree. Total scores range from 10 to 40 points. The reliability index calculated for Spanish adults is 0.86 [49].

#### 2.3.8. Satisfaction with Life

Diener’s [50] Satisfaction with Life Scale (SWLS) was used to measure overall satisfaction with a person’s life, in the version validated in Spanish by Atienza et al. [51]. The reliability of this scale calculated by Cronbach’s alpha coefficient was 0.836 [52]. This tool analyses global cognitive assessments of satisfaction with one’s own life through five items, with responses on a five-point Likert scale ranging from strongly disagree (1) to strongly agree (5). Final scores range from five to 35 points. Higher scores indicate greater life satisfaction.

#### 2.3.9. Perceived Stress

The Perceived Stress Scale (PSS) measured students’ perceived stress using the Spanish version [53]. The reliability of the shorter Spanish version was adequate, α = 0.82. This scale probes into feelings and thoughts experienced during the month prior to the survey. The scale has 14 items that are scored on a five-point Likert scale (0 = never; 4 = very often) based on how often they felt that way. Total scores are obtained, after recoding certain items, by adding them all together, producing results in the range of zero to 56 points. Higher scores indicate greater levels of perceived stress.

#### 2.3.10. Suicidal Behaviour

The SENTIA-Breve Scale [54], validated in Spanish young people, assessed suicidal behaviour. It contains five statements related to individuals’ thoughts and feelings during the six months prior to the survey, in order to determine the general factor of suicidal behaviour and the specific ones, i.e., suicidal act or planning, communication, and ideation. Responses are dichotomous, i.e., yes = 0 and no = 1. All values are added together, resulting in total scores ranging from zero to five points, with higher scores indicating greater severity or risk of suicide. Reliability was equal to 0.97 Ω.

Additionally, the survey tool included randomly interleaved question pairs from the Oviedo Infrequency Scale (INF-OV) [33] as part of the compilation of all other measures. These questions detect respondents who provide random, pseudo-random, or dishonest responses on the overall survey tool. There are twelve self-report items that are designed to have an obvious correct answer among dichotomous response options (0 = yes; 1 = no). Students who provided two or more counterintuitive responses on this scale were removed from further analysis. A total of 14 participants were excluded on this basis.

### 2.4. Statistical Analysis

The first step was to analyze the quantitative variables based on means and standard deviations, and the qualitative variables according to frequencies. Subsequently, the normality and homoscedasticity of the data for all variables were assessed using the Kolmogorov–Smirnov test with Lilliefors correction and the Levene test. The data for all variables were found to have a non-normal distribution.

Since the data did not meet the assumption of normality, non-parametric tests were employed. In particular, the Kruskal–Wallis test was used to compare differences between groups. Additionally, Spearman’s correlation coefficients were applied to assess the relationships between variables, as this test measures associations without assuming a normal distribution, making it more suitable for the type of variables analyzed in this study.

Finally, multiple linear regression was conducted to identify the factors associated with adherence to the Mediterranean Diet (MD). Despite the observed non-normality, this analysis is justified because multiple linear regression is robust to moderate deviation from normality, especially with large sample sizes such as in this study (n = 1268). Furthermore, the assumptions of multicollinearity, homoscedasticity, and linearity were verified to ensure the validity of the results. The model included variables such as perceived stress, suicidal behaviour, self-esteem, life satisfaction, emotional and behavioural difficulties, emotional intelligence, physical activity, sedentary lifestyle, alcohol consumption, and compulsive internet use. The backward elimination method was used, allowing the model to be refined by progressively removing non-significant variables while retaining those with *p*-values < 0.05 in the final model. This technique was chosen for its effectiveness in identifying the most relevant predictors in exploratory studies.

The collected data were analyzed using IBM SPSS Statistics version 29, with statistical significance set at *p* < 0.05.

## 3. Results

Table 1 presents adherence to the Mediterranean diet (MD) based on various sociodemographic factors. The results revealed significant differences in adherence according to age and income level. Regarding age, adults over 26 years old exhibited higher rates of high adherence to the MD (36.8%) compared to emerging adults aged 17 to 25 years (27.4%) (*p* = 0.014). Similarly, adherence to the MD showed significant differences based on income level, with individuals earning ≥ 2000 euros displaying the highest rates of high adherence (38.1%), compared to those with incomes between 0 and 999 euros (27.6%) and 1000 and 1999 euros (33%) (*p* = 0.023). No significant differences were found in adherence to the MD based on sex (*p* = 0.742).

Table 2 shows the results of four mental well-being indicators such as perceived stress, suicidal behaviour, self-esteem, and life satisfaction, depending on adherence to the MD. It specifically shows how students with high adherence to the MD had lower rates of perceived stress and suicidal behaviour, as well as higher self-esteem and life satisfaction.

Table 3 presents the results on emotional and behavioural difficulties, as well as the subscales and components of emotional intelligence, based on the level of adherence to the Mediterranean Diet (MD). Students with high adherence to the MD reported significantly lower levels of emotional problems (*p* < 0.001), behavioural problems (*p* = 0.033), hyperactivity (*p* < 0.001), and peer conflicts (*p* < 0.001). Conversely, students with high adherence to the MD exhibited greater prosocial behaviour (*p* = 0.043).

Regarding emotional intelligence, the results indicated that students with higher adherence to the MD obtained significantly higher scores across all assessed dimensions, including mindfulness (*p* = 0.002), emotional clarity (*p* < 0.001), and emotional repair (*p* < 0.001).

Table 4 presents lifestyle habits according to the level of adherence to the Mediterranean Diet (MD). Students with high adherence to the MD reported significantly higher levels of physical activity (*p* < 0.001) and lower weekly sedentary time (*p* = 0.002). Additionally, compulsive internet use was lower among students with high adherence (*p* < 0.001). No significant differences were found in alcohol consumption across adherence levels (*p* = 0.131).

Finally, Table 5 shows the results of the multiple linear regression related to adherence to the MD. The results reveal that higher adherence to the Mediterranean Diet (MD) is associated with higher levels of emotional intelligence and physical activity, as well as lower rates of suicidal behaviour, emotional problems, and compulsive internet use. The multiple linear regression model explained 7.7% of the variance in adherence to the Mediterranean Diet (adjusted R^2^ = 0.077).

## 4. Discussion

According to the scores obtained in the KIDMED questionnaire, 29.26% of university students in this study showed high adherence to the MD, 54.65% had moderate adherence, and 16.09% had low adherence. When comparing these results with studies conducted on Spanish university students, the percentages of high adherence in this study are lower [55,56]. Nevertheless, the high adherence values recorded in this study exceed those reported in university students from other Mediterranean countries, such as Cyprus (26.9%) [57], Turkey (9.4%) [58], Portugal (12.50%) [59], and Italy (22.7%) [60]. Antonopoulou et al. [14] note that university students have been moving away from healthy eating patterns, especially the Mediterranean Diet. This is even more evident among those living in Mediterranean countries, notably when living away from the family home. Furthermore, results from this study indicate that adherence to the MD is linked to some sociodemographic factors, a number of mental and emotional health indicators and other lifestyle habits.

The adherence to the MD in these university students revealed significant differences according to age and income level. There is a direct relationship between both variables, i.e., the higher the age and socio-economic status, the higher the adherence to the MD. On the one hand, these results are consistent with previous research showing that age is a protective factor for adherence to the MD, in particular, those who live in or close to their parents’ home [16,61]. The young adult group has the lowest levels of adherence to MD during the first year of university [25,62]. On the other hand, the economic effort required for good adherence to the DM is high [63]. This can be a barrier for people of low socio-economic status. One such group is young people, who tend to have a lower income level than adults, as they often do not have a stable source of income or a stable job [64,65]. Mendonça et al. [66] conclude that the risk profile for low adherence to the MD consists of being young and having a low socio-economic status.

In terms of mental health, university students with high adherence to the MD showed lower levels of perceived stress. These results are in line with previous research that has documented the benefits of this dietary pattern on mental health, both in the United States and in Mediterranean countries such as Italy, Spain, and Greece, as well as in other European Union countries and Australia [5]. Carvalho et al. [67], in a study with a Brazilian population, indicate that adherence to the MD is inversely associated with cortisol levels, which is a key hormone in the stress response, suggesting that healthy eating patterns may have a protective effect against future mental health problems. Furthermore, research conducted by Antonopoulou et al. [14], of studies carried out exclusively with university students from Mediterranean countries, highlights that higher perceived stress is associated with lower intake of fruits and vegetables, which are rich in antioxidants and anti-inflammatory substances that help reduce stress levels [68,69]. Similarly, polyphenol-rich foods, present in fruits, vegetables, and olive oil, play a key role in neuroplasticity and neurotransmitter modulation, such as serotonin and dopamine, both of which are crucial for mood regulation and stress perception [69,70].

These factors could also explain the findings in relation to self-esteem and life satisfaction, where students with higher adherence to the MD exhibited higher indices. The fact that these students report higher levels of self-esteem and life satisfaction is in line with studies relating diet quality to the psychological well-being of university students from Mediterranean countries [14]. Taking a comprehensive approach, a healthy diet can positively influence various aspects of health, such as body perception, physical well-being, cognitive performance, and reduced risk of chronic diseases, including cardiovascular diseases [71]. In turn, these factors impact general well-being, health perception, and self-esteem, both the international population [72] and the Spanish university students [73]. Likewise, research by Biasini et al. [74] with Italian adults, has shown that adherence to a healthy diet is associated with better self-perception, which could translate into higher self-esteem and life satisfaction. This link between a healthy diet and self-perception supports the idea that eating habits influence not only physical health but also psychological well-being and self-image.

Students with high adherence to the MD have lower reported suicidal behaviour, which was also observed in the regression analysis. Previous research has highlighted the relationship between diet quality and emotional health, through mechanisms such as neuroinflammation, neurogenesis, or synaptic plasticity [75,76]. The Mediterranean Diet features a high consumption of plant-based foods and fish, reduced sugar and processed and red meat intake, and the use of olive oil as the main vegetable fat source. These elements are associated with an improvement in endothelial function, an increase in eicosanoid levels and the synthesis and regulation of serotonin, which appears to have a protective effect against depression [77]. Thus, anti-inflammatory diets such as the Mediterranean Diet may reduce the risk and symptoms of depression, a key factor related to suicidal ideation [78]. Previous studies with adults from around the world, specifically indicate that fruit and vegetable consumption is associated with increased optimism and sense of efficacy, which may decrease the risk of psychological distress or depression, the latter being a major risk factor for suicide [68]. Specifically, in the Korean adult population, a 15% increased likelihood of suicidal ideation was observed among individuals who did not consume fruits and vegetables, in comparison to those who consumed them [79].

Students with high adherence to the MD had lower overall rates of emotional and behavioural difficulties, as well as lower scores on “emotional problems”, “behavioural problems”, “hyperactivity”, and “peer conflicts” components. However, these students showed significantly higher values in the “prosocial behaviour” category. These findings concur with previous studies in the Region of Murcia (Spain), that associate the MD with reduced behavioural and emotional problems, as well as increased prosocial behaviour [80]. Previous studies in Europe, Asia and Oceania suggest that healthy dietary patterns, featuring fruit, vegetables, and whole grains, have a protective effect against hyperactivity [81]. Moreover, diets deficient in essential nutrients are associated with an increased risk of emotional and behavioural problems in children and adolescents, whereas healthy eating behaviours, such as the Mediterranean Diet, favour emotional stability, and behavioural control [10,82,83]. The systematic review of Bozzatello et al. [84], Omega-3 fatty acids found in foods such as fish particularly improve brain health and the regulation of key neurotransmitters, such as serotonin, which helps to reduce symptoms of depression and anxiety.

Compared to the MD, unhealthy diets accelerate ageing, increase the risk of cardiometabolic diseases, cause neuroinflammation, and neuroanatomical alterations and contribute to the onset of anxiety and even social isolation. This indicates that the Mediterranean diet could be an effective therapeutic approach against psychosocial stress [85]. By way of example, a longitudinal study in Japanese children found that vegetable consumption may have a protective effect against behavioural problems and promote prosocial behaviour [86]. In addition, regression analysis identified a negative association between adherence to the MD and emotional problems. Studies in adolescents have shown that emotional symptoms are more frequent in those who follow a dietary pattern rich in sweets and fats and have low adherence to the MD [87]. In keeping with these results, a study conducted with university students found that higher adherence to the Mediterranean Diet is negatively associated with anxiety, stress, emotional problems, and negative emotions [88].

Students with higher adherence to the MD scored higher on the dimensions of mindfulness, clarity and emotional repair in relation to emotional intelligence. These results were supported by regression analysis, which found positive associations with the mindfulness aspect. Emotional intelligence, understood as the ability to perceive, understand and appropriately manage emotions, facilitates psychological well-being, reduces negative emotions and promotes the adoption of healthy behaviours [89]. Similarly, low levels of emotional intelligence have been found to be directly related to the adoption of unhealthy habits, such as low adherence to the MD, physical inactivity, and alcohol and tobacco consumption in Italian university students [90]. The regression analysis results reinforce the hypothesis that greater development of emotional intelligence improves the ability to manage and process complex emotions, which is essential for maintaining healthy habits, especially in high-stress contexts, such as academia [91].

As for lifestyle habits, students with greater adherence to the MD had a higher number of METS (metabolic energy expenditure). This finding is consistent with earlier studies in Spanish adolescents, such as Grao-Cruces et al. [92], who found a direct association between adherence to the MD and higher levels of physical activity, as well as lower levels of sedentary behaviour. The MD provides a sustained source of energy and nutrients needed to optimise physical and mental performance [93]. Roya et al. [94] point out that physically active young people require higher energy expenditure, which implies a higher intake of essential nutrients such as carbohydrates, vitamins, and quality protein. Additionally, one possible explanation for these findings is that students who follow the MD may have higher health literacy, which directly influences the adoption of healthy lifestyle behaviours [95].

Lastly, students with high adherence to the MD showed lower compulsive internet use, which was also corroborated by regression analysis. Previous studies with adolescents in the Mediterranean region confirm that internet addiction has a negative impact on maintaining a healthy lifestyle [96]. In particular, research conducted with university students selected from four different universities in Pakistan revealed that those with problematic internet use have a poorer diet quality, marked by lower consumption of fruits and vegetables, skipping meals, snacking, carbonated soft drinks, and fast food consumption [97]. Similarly, internet addiction appears to be linked to unhealthy eating patterns, such as skipping meals while browsing the internet, increased processed and easily accessible food consumption, as well as alterations in body image perception resulting from the use of social media, which contribute to the development of body image disorders [98,99]. Furthermore, other studies with Turkish university students suggest that the duration of internet use influences smartphone and internet addiction, which has a direct impact on eating disorders in students [100].

This study has certain limitations. Firstly, this is an online survey carried out with a specifically selected sample and the results cannot be extrapolated to the general population. However, they do offer an insight into the situation among university students in the north of Spain. Secondly, the study is based on self-report, so the usual limitations of self-report survey items, such as bias induced by subjectivity, must be taken into account. Nevertheless, the tools used have been shown to be reliable and valid in previous studies with similar populations. Thirdly, due to the breadth of the survey and the need to minimise the time required to complete it, there are data elements that have not been collected, such as their previous health status. Finally, the cross-sectional nature of this research precludes conclusions about causality. This should be addressed through future longitudinal studies. In terms of future research, the findings suggest the need for longitudinal follow-up of students to monitor improvements throughout the university period and at different points in their academic career.

## 5. Conclusions

The results show associations between adherence to the MD and various health, mental and emotional well-being indicators in university students, as well as age and income level. A high adherence to the MD was found in 29.26% of students, which highlights the need to promote healthy eating habits during university to improve both present and future health. Higher levels of emotional intelligence, as well as lower rates of suicidal behaviour, emotional problems and compulsive internet use, were associated with higher rates of adherence to the MD, accounting for 7.7% of the variance.

The observed associations emphasise the need to design and implement measures to improve adherence to the MD in the university setting. These strategies could include the creation of nutritional education and literacy programmes, the provision of an adequate supply of healthy foods in the educational environment, and the promotion of other healthy habits such as physical activity. In addition, integrating Mediterranean dietary patterns into campus meal programs could facilitate healthier food choices by ensuring the availability of nutrient-dense options, such as fruits, vegetables, whole grains, legumes, and healthy fats like olive oil. These initiatives could enhance adherence to the MD and, therefore, have a positive impact on university students’ mental and emotional health and lifestyles. Moreover, universities could implement educational campaigns and workshops aimed at increasing awareness of the benefits of the MD for both physical and mental well-being. Collaborations between university health services, student organisations, and nutrition experts may also support personalised nutritional counselling, fostering healthier eating habits within the student population.

## Figures and Tables

**Table 1:** Adherence to the Mediterranean Diet according to various sociodemographic factors.

			Low Adherence	Medium Adherence	High Adherence	*p* Value
			%	%	%	
Age	Emerging adults (17–25 years)	1021	16.50%	56.10%	27.40%	0.014
	Adults (>26)	247	14.60%	48.60%	36.80%	
Sex	Female	823	15.60%	55.20%	29.20%	0.742
	Male	445	17.10%	53.50%	29.40%	
Incomes (euros)	0–999	964	17.40%	55.00%	27.60%	0.023
	1000–1999	212	14.20%	52.80%	33.00%	
	≥2000	92	6.50%	55.40%	38.10%	

**Table 2:** Mental well-being indicators based on adherence to the Mediterranean Diet.

	Low Adherence (n = 204)		Medium Adherence (n = 693)		High Adherence (n = 371)		*p* Value
	M	SD	M	SD	M	SD	
Perceived stress (PSS)	29.65	9.69	27.64	8.49	25.23	8.48	<0.001
Suicidal behaviour (SENTIA)	0.74	1.34	0.47	1.05	0.29	0.84	<0.001
Self-esteem (Rosemberg)	29.59	6.40	30.96	5.89	32.92	5.62	<0.001
Life satisfaction (SWLS)	15.75	4.18	17.25	3.92	17.85	4.01	<0.001

Note: M = mean; SD = standard deviation.

**Table 3:** Emotional intelligence, emotional, and behavioural difficulties depending on adherence to the MD.

	Low Adherence (n = 204)		Medium Adherence (n = 693)		High Adherence (n = 371)		*p* Value
	M	SD	M	SD	M	SD	
Emotional and behavioural difficulties (SDQ)	14.11	5.90	12.61	5.30	10.80	4.80	<0.001
Emotional problems	4.69	3.13	4.08	2.75	3.21	2.60	<0.001
Behavioural problems	2.26	1.38	2.10	1.37	1.94	1.26	0.033
Hyperactivity	4.35	2.21	3.91	2.07	3.47	2.18	<0.001
Peer conflicts	2.82	1.78	2.52	1.60	2.18	1.47	<0.001
Prosocial	8.16	1.83	8.45	1.57	8.59	1.50	0.043
Emotional Intelligence (Mindfulness) (TMMS)	24.27	7.44	26.29	6.77	25.74	6.73	0.002
Emotional Intelligence (Clarity) (TMMS)	22.12	6.82	24.72	6.52	25.68	6.75	<0.001
Emotional Intelligence (Remediation) (TMMS)	23.78	6.56	24.85	6.33	26.74	6.37	<0.001

Note: M = mean; SD = standard deviation.

**Table 4:** Lifestyle Habits Based on Adherence to the Mediterranean Diet.

	Low Adherence (n = 204)		Medium Adherence (n = 693)		High Adherence (n = 371)		*p* Value
	M	SD	M	SD	M	SD	
Physical Activity (METS)	1727.51	1630.39	2437.19	2207.55	3511.33	2617.76	<0.001
Weekly sedentary time (minutes)	451.24	234.45	393.41	207.80	387.78	176.72	0.002
Alcohol consumption (AUDIT)	4.03	4.18	3.72	3.81	3.20	3.25	0.131
Compulsive internet use (CIUS)	19.90	11.13	17.15	10.63	14.11	10.47	<0.001

Note: M = mean; SD = standard deviation.

**Table 5:** Multiple linear regression related to adherence to the MD.

	B	Error Deviation	Standardised Beta	t	*p* Value	Adjusted R^2^
Suicidal behaviour (SENTIA)	−0.204	0.071	−0.085	−2.866	0.004	0.077
Emotional Intelligence (Mindfulness)	0.035	0.010	0.096	3.408	0.001	
Emotional problems (SDQ)	−0.077	0.015	−0.164	−5.249	<0.001	
Compulsive internet use (CIUS)	−0.031	0.007	−0.132	−4.456	<0.001	

Note: B = Beta Regression coefficient; Adjusted R^2^: coefficient of determination. Multiple linear regression model using the backward elimination method. Variables included in the model were: perceived stress, suicidal behaviour, self-esteem, life satisfaction, Emotional and behavioural difficulties subscales (SDQ), emotional intelligence (attention), emotional intelligence (clarity), emotional intelligence (repair), physical activity, sedentary lifestyle, alcohol consumption, and compulsive internet use (CIUS).

## Data Availability

Data are contained within the article.

## Abbreviations

The following abbreviations are used in this manuscript:

MD	Mediterranean Diet
